# Leptin gene promoter DNA methylation in WNIN obese mutant rats

**DOI:** 10.1186/1476-511X-13-25

**Published:** 2014-02-05

**Authors:** Rajender Rao Kalashikam, Padmavathi JN Inagadapa, Anju Elizabeth Thomas, Sugeetha Jeyapal, Nappan Veettil Giridharan, Manchala Raghunath

**Affiliations:** 1 Molecular Genetics, National Center for Laboratory Animal Sciences, National Institute of Nutrition, Jamai Osmania P O, Hyderabad 500 007, India; 2 Division of Endocrinology and Metabolism, National Institute of Nutrition, Hyderabad, India

**Keywords:** Leptin expression, Promoter DNA methylation, Insilico analysis, Obesity, WNIN

## Abstract

**Background:**

Obesity has become an epidemic in worldwide population. Leptin gene defect could be one of the causes for obesity. Two mutant obese rats WNIN/Ob and WNIN/GROb, isolated at National Centre for Laboratory Animal Sciences (NCLAS), Hyderabad, India, were found to be leptin resistant. The present study aims to understand the regulatory mechanisms underlying the resistance by promoter DNA methylation of leptin gene in these mutant obese rats.

**Methods:**

Male obese mutant homozygous, carrier and heterozygous rats of WNIN/Ob and WNIN/GROb strain of 6 months old were studied to check the leptin gene expression (RT-PCR) and promoter DNA methylation (MassARRAY Compact system, SEQUENOM) of leptin gene by *invivo* and *insilico* approach.

**Results:**

Homozygous WNIN/Ob and WNIN/GROb showed significantly higher leptin gene expression compared to carrier and lean counterparts. Leptin gene promoter DNA sequence region was analyzed ranging from transcription start site (TSS) to-550 bp length and found four CpGs in this sequence among them only three CpG loci (-309, -481, -502) were methylated in these WNIN mutant rat phenotypes.

**Conclusion:**

The increased percentage of methylation in WNIN mutant lean and carrier phenotypes is positively correlated with transcription levels. Thus genetic variation may have effect on methylation percentages and subsequently on the regulation of leptin gene expression which may lead to obesity in these obese mutant rat strains.

## Introduction

Obesity has become an alarming epidemic to global health with increased hypertension, coronary atherosclerosis, myocardial hypertrophy, type II diabetes and cardiovascular morbidity & mortality. Although obesity is caused by both genetic and environmental factors the molecular mechanism underlying obesity is not yet well established. It is certainly a multifaceted disease with complex interactions between genetic traits and environmental factors, regulated by epigenetic mechanisms.

Earlier many experiments have been conducted to study obesity and the underlying mechanisms involved in various animal models especially in Ob/Ob and db/db mouse strains, Zucker and Koletsky rat strains. Two mutant obese rat strains were isolated from WNIN stock, designated as WNIN/Ob and WNIN/GROb in 1994, at National Institute of Nutrition, Hyderabad, India [1]. These mutant rats are characterized by hyperphagia, hyperinsulinemia, hypertriglyceridemia, higher body fat% (47%) and are found to be leptin resistant as well [1]. Additionally WNIN/GROb rat show impaired glucose tolerance (IGT) too. The inheritance of obesity in these rats follows a co-dominant pattern as per Mendelian laws and the phenotypic ratio of the offspring is 1:2:1 (1 lean^+/+^ : 2 carrier^+/-^ : 1 Obese^-/-^). Both the homozygous obese male and females are infertile while the carriers are fertile and are used for breeding and maintaining the stock.

Interest on the pathophysiology of obesity has been intensified recently with the discovery of leptin [2]. Leptin, a 167 amino acid protein and the product of the *obese* gene *Leptin (LEP),* is a key hormone primarily secreted by adipocytes [3]. Leptin levels increase exponentially with increasing fat mass [4,5]. Leptin inhibits food intake and regulates energy intake and expenditure by central action on the hypothalamus. Activation of central leptin receptors increases the activity of the sympathetic nervous system [6,7] which stimulates energy expenditure in adipose tissue [8,9]. Leptin levels significantly increase in obese humans and this endogenous hyperleptinemia does not reduce appetite or increase energy expenditure suggesting that they are leptin resistant. Recent studies demonstrate that leptin promoter methylation regulates leptin expression [10]. Melzner et al reported that CpGs in the proximal leptin promoter were highly methylated in pre-adipocytes and gets highly demethylated as they mature to terminally differentiated adipocytes. This suggests that the methylation of CpGs inhibits leptin expression, while their demethylation activates the expression [11]. Although WNIN/Ob rats and WNIN/GROb were found to be leptin resistant like other rodent models the regulatory mechanisms underlying this resistance is not yet known. Thus the present study aims to understand the leptin resistance by determining the promoter DNA methylation pattern of leptin gene which presumably should explain the extent of leptin expression seen in these animals.

## Methods

### Animal study

The experimental procedure was approved by the ethics committee on animal experiments at the National Institute of Nutrition (NIN), Hyderabad, India and was performed in accordance with the principles of laboratory animal care. Male obese WNIN rats i.e.homozygous, WNIN/Ob obese (-/-), heterozygous WNIN/Ob carrier (+/-), homozygous WNIN/Ob lean (+/+) and WNIN/GROb obese (-/-), WNIN/GROb carrier (+/-), WNIN/GROb lean (+/+) of 6 months old (3 per strain) were obtained from National Centre for Laboratory Animal Sciences (NCLAS), NIN, Hyderabad, India. They were housed individually in polypropylene cages with wire mesh bottom cases maintained at 22 ± 2°C, under standard lighting conditions (12-h light/dark cycle).Animals were fed *ad libitum* with stock rat chow and free access to deionized water. The animals were sacrificed and their retroperitoneal adipose tissue was collected and snap frozen in liquid nitrogen and stored at -80°C until analysis.

### Leptin mRNA expression in retroperitoneal adipose tissue by semi-quantitative PCR

Total RNA was isolated from 100 mg of adipose tissue (retroperitoneal) using Qiazol reagent. This was followed by the synthesis of cDNA from 2 μg of total RNA using Invitrogen kit (Invitrogen Life technologies, Carlsbad, CA). Primers were designed with the aid of primer quest software (Integrated DNA Technologies, Corolville, IOWA). Semi-quantitative PCR was conducted to analyze the expression of leptin (5′ACTTCATTCCCGGGCTTC3′; 5′GGTCTCGCAGGTTCTCCA3′), with the internal standard18SrRNA (5′CCAGAGCGAAAGCATTTGCCAAGA3′;5′AATCAACGCAAGCTTATGACCCGC3′). The amplified products were resolved on 1.2% agarose gel electrophoresis and the image was quantified with the Bio-Rad gel documentation system using Quantity One software (Bio-Rad Laboratories, Herculus, CA). Results were expressed as the ratio of the intensities of the band of the gene of interest to that of the 18s rRNA.

### DNA isolation

DNA was isolated from 100 mg of retroperitoneal adipose according to the manufacturer’s instructions of QIAGEN DNeasy tissue kit (Qiagen India Pvt. Ltd., India). The isolated gDNA was quantified using a ND-1000 spectrophotometer (Nano Drop Technologies, Inc).

### Sodium bisulfite modification

Bisulfite modified gDNA was prepared using the EZ DNA Methylation-Gold kit (Zymo Research, catalog #D5005) according to the manufacturer’s instructions. The bisulfate reaction was performed on 0.5 μg gDNA and the reaction volume was adjusted to 20 μl with sterile water and 130 μl of CT conversion reagent was added. The sample tubes were placed in a thermal cycler (Bio-Rad) and the following steps were performed: 10 min at 98°C, 2 h 30 min at 64°C and stored at 4°C. The converted samples were added into Zymo-Spin IC^TM^ Column containing 600 μl of the M-Binding Buffer and mixed by inverting the column several times. After mixing, the column was centrifuged at full speed for 30 sec and discarded the flow-through. The column was then washed by adding 200 μl of M-Desulphonation buffer and incubated at room temperature (20-30°C) for 15-20 min. After incubation, the column was centrifuged at full speed for 30 sec. The column was again washed by adding 200 μl of M-Wash buffer and spun at full speed (this step was repeated). The converted gDNA was eluted by adding 20 μl of M-Elution buffer into the column and spin. The bisulfite converted gDNA samples were stored at -20°C until methylation analysis. Pyro Q-CpG Software (Biotage) was used to check automatically the generation of dispensation order (sequence) and it also performs quality control of each sample for completion of bisulfite treatment, eliminating the need for manual inspection and estimation levels of non-converted DNA.

### Quantitative methylation analysis

Quantitative methylation analysis of the leptin gene promoter was performed with the MassARRAY Compact system (Sequenom, San Diego, CA, USA). The target regions of the sodium bisulfate treated gDNA were amplified by PCR with a set of primers: leptin_10 left primer 5′TTATGGATTAGTAATGAAGATTTTAATAGA3′; leptin_10 right primer 5′TCAATAAAATACCCACCCTTAAAAA3′. Each forward primer contained a 10-nucleotide tag (5′-AGGAAGAGAG-3′) to balance the PCR, and each reverse primer contained a T7 promoter tag and sequence insert (5′-CAGTAATACGACTCACTATAGGGAGAAGGCT-3′) for in vitro transcription. The amplification protocol comprised an initial incubation at 94°C for 4 min; 45 cycles of denaturation at 95°C for 20 s, annealing at 62°C for 30 s, and extension at 72°C for 1 min; and a final incubation at 72°C for 3 min. Unincorporated deoxynucleoside triphosphates were dephosphorylated by the addition of 2 μl of Premix for in vitro transcription including 0.3 U of shrimp alkaline phosphatase (Sequenom, San Diego, CA, USA). The reaction mixture was incubated at 37°C for 40 min, after which the phosphatase was inactivated by incubation for 5 min at 85°C. A portion of the PCR products (2 μl) was then subjected to in vitro transcription, with RNase A cleavage being used for the T-reverse reaction (Sequenom). Samples were spotted onto a 384-pad Spectro-CHIP (Sequenom) with the use of a MassARRAYnanodispenser (Sequenom) and spectra were acquired with a MassARRAY compact matrix-assisted laser desorption ionization time-of-flight (MALDI-TOF) mass spectrometer (Sequenom). The resulting methylation calls were analyzed with EpiTyper software (Sequenom) to generate quantitative results for each CpG site or an aggregate of multiple CpG sites.

### Insilico analysis of leptin promoter

The leptin gene promoter DNA sequence of rat was obtained from the UCSC genome browser (http://genome.ucsc.edu/). In this study the promoter was defined as the one which can extend from transcription starting site (TSS) to -550bp upstream. The CpG island searcher (http://cpgislands.usc.edu) was used to screen for CpG islands (D.T and P.A.J). The methylator software (http://bio.dfci.harvard.edu/Methylator) was used to identify CpG and predict whether CpG in a promoter DNA sequence are likely to be methylated or not.

### Statistical analysis

All values were represented as mean ± S.E. Data was analyzed by one-way analysis of variance (ANOVA) followed by the multiple range test or least significant difference method. Wherever heterogeneity of variance was observed, differences between groups were tested using non-parametric Mann-Whitney U test. The differences were considered significant at p < 0.05.

## Results

### Insilico analysis of leptin gene promoter DNA sequence

The rat leptin gene promoter DNA sequence (TSS to -550bp) showed 118 C counts (19.6%), 144 G counts (24%) and 5 GC counts (0.8%) and the odds ratio was 17.6%. There were no CGIs predicted in this 550bp length upstream DNA sequence while four CpGs were identified (Table 1). Among the identified CpGs only two CpGs were predicted to be methylated. The closest methylated CpG locus was noticed at -309bp distance from the TSS and the other methylated site was located at -502bp away from TSS.

**Table 1:** **The leptin promoter DNA sequence *in silico *** **analysis - The CpG locus and predicted methylated CpG sites**

**S.No**	**Promoter length (bp)**	**No. of CpG**	**Position of CpG**	**No. of methylated CpG**	**Methylated CpG position**
1	TSS to -100	0	0	0	0
2	-101 to -200	0	0	0	0
3	-201 to -300	0	0	0	0
4	-301 to -400	1	-309	1	-309
5	-401 to -500	2	-447, -481	1	0
6	-501 to -550	1	-502	1	-502

### Leptin gene expression in adipose tissue

**Figure 1:**
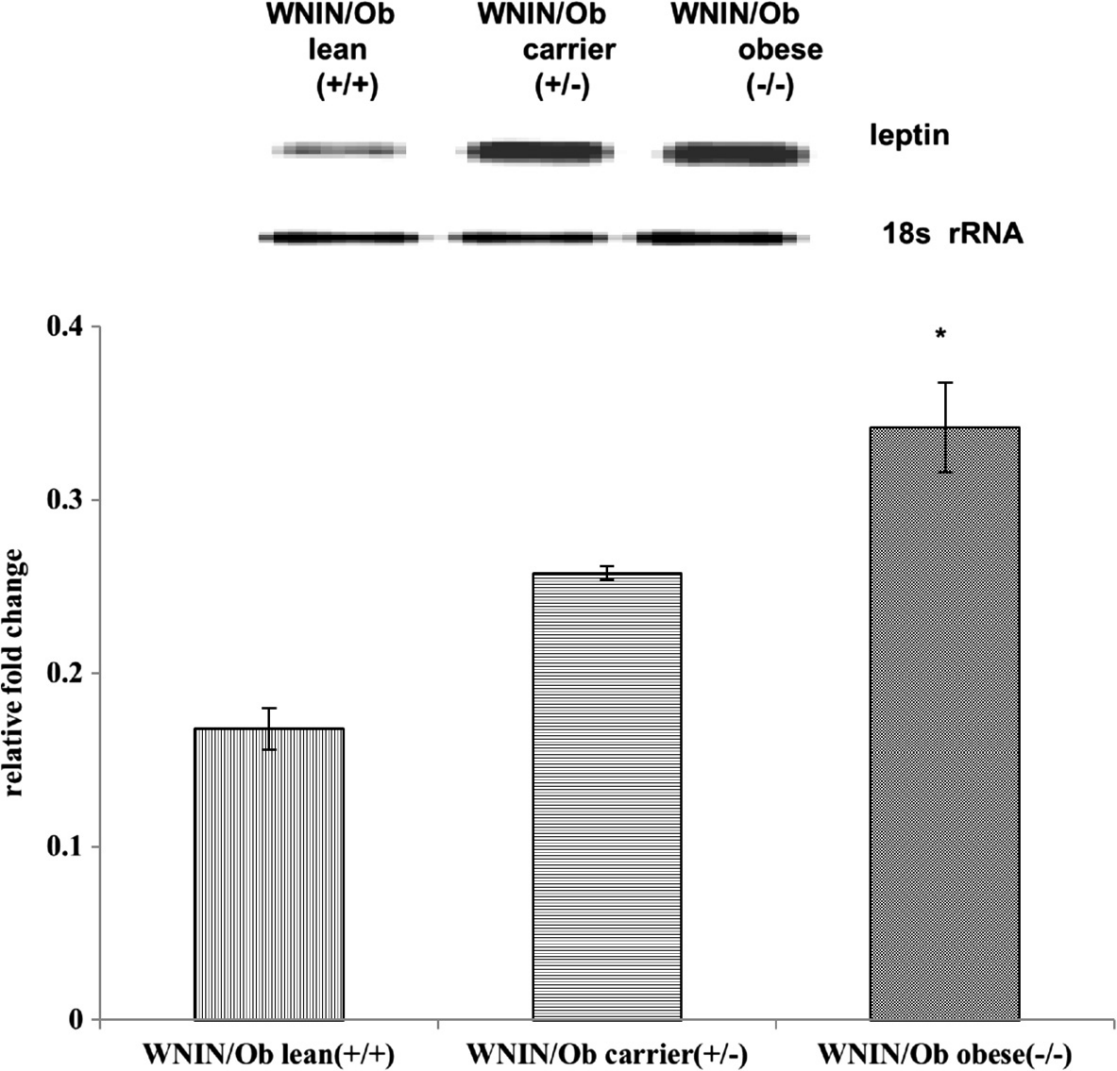
Leptin gene expression in adipose tissue of WNIN/Ob rats.

**Figure 2:**
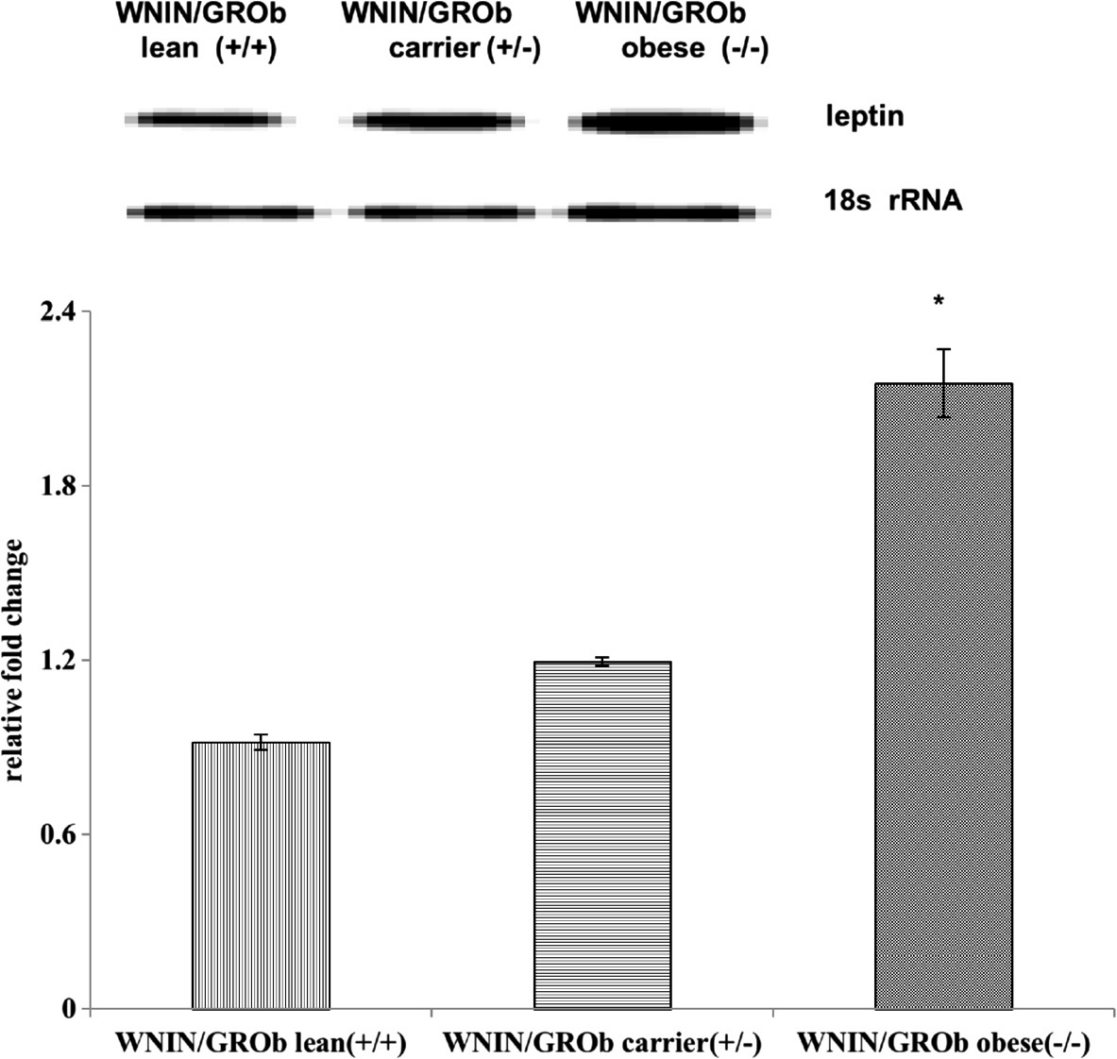
Leptin gene expression in adipose tissue of WNIN/GROb rats.

Reverse transcription-PCR experiments were carried out in visceral adipose tissue to evaluate the leptin mRNA expression. Homozygous Ob/Ob was found to have maximum leptin mRNA expression followed by carrier and lean in that order. The semi-quantitative analysis of leptin mRNA expression was found to be significantly (p < 0.05) decreased in WNIN/Ob lean and WNIN/Ob carrier rats when compared to their WNIN/Ob obese phenotypes (Figure 1). Similarly, a significant decreased leptin mRNA expression was also depicted in WNIN/GROb lean and carrier rats when compared with their homozygous obese rats (Figure 2). Interestingly, the leptin mRNA expression levels were higher in all the three phenotypes of WNIN/GROb when compared with their WNIN/Ob rats. The percentage of relative mRNA expression was higher in WNIN/GROb rats compared with WNIN/Ob rats. The similar trend was noticed between WNIN/Ob carrier Vs WNIN/GROb carrier and WNIN/Ob lean Vs WNIN/GROb phenotypes.

### Leptin gene promoter DNA methylation

**Figure 3:**
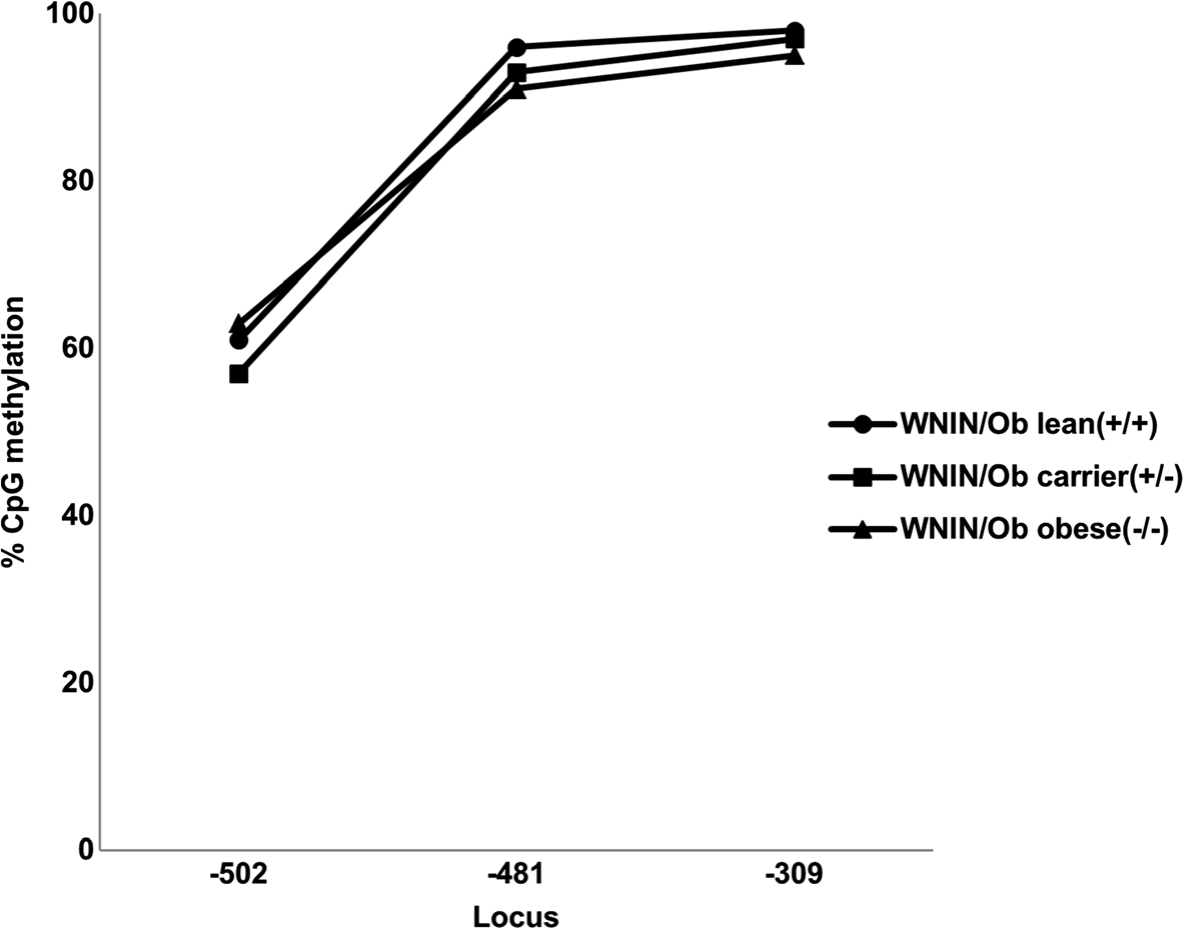
Leptin gene promoter DNA methylation in WNN/Ob rats.

Leptin gene promoter DNA sequence region was analyzed ranging from TSS to – 550 bp length. It was found that among the four CpGs present in this sequence only three CpG loci were methylated in these WNIN mutant rat phenotypes. The percentages of leptin promoter DNA methylation at three different loci -309, -481 and -502 were noted in WNIN/Ob and WNIN/GROb phenotypes. It was observed that the methylation percentage was less in WNIN/Ob obese rats (95%) compared to their carrier (97%) and lean (98%) counterparts at the locus -309. Similarly the methylation percentage was less in WNIN/Ob obese rats (91%) compared to their carrier (93%) and lean (96%) counterparts at the locus -481. However the methylation percentage was more in WNIN/Ob obese rats (63%) compared to their carrier (57%) and lean (61%) phenotypes at the locus -502 (Figure 3).

**Figure 4:**
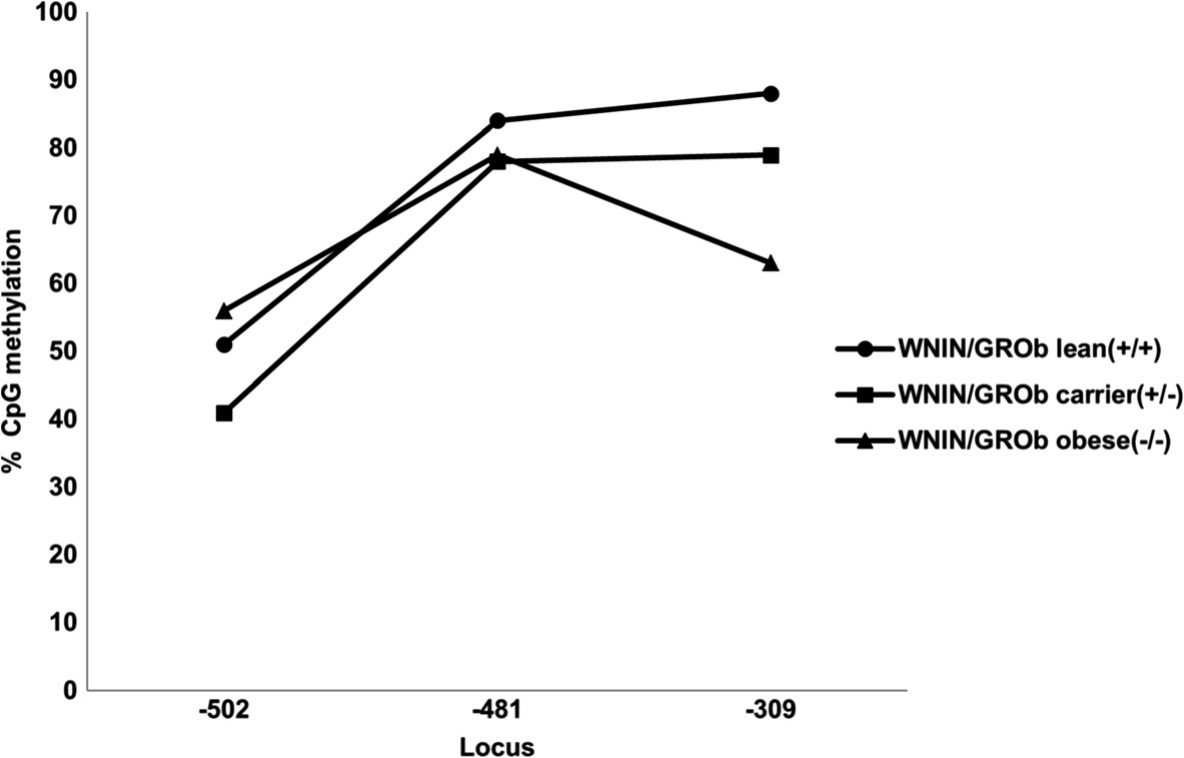
Leptin gene promoter DNA methylation in WNN/GROb rats.

**Figure 5:**
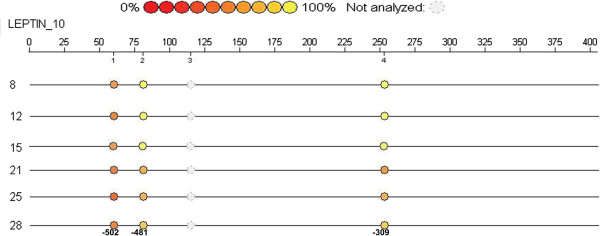
Epigram of leptin gene promoter in WNIN/Ob and WNIN/GROb rats.

Similarly in WNIN/GROb phenotypes, the percentage of leptin promoter DNA methylation was lower in obese phenotype (63%) at the locus -309 when compared to their carrier (79%) and lean rats (88%). Although the methylation percentage in obese rats (79%) was comparable with that of carrier phenotypes (78%), it was found to be lower in obese compared to lean rats (84%) at the locus -481. However the methylation percentage was higher in obese phenotypes (56%) followed by lean (51%) and carrier rats (41%) at the third locus -502 (Figure 4). It was also observed that the methylation percentages were found to be relatively more in WNIN/GROb mutant phenotypes compared to WNIN/Ob phenotypes at the locus -309 and -482 while the trend was similar in the percentage of methylation in both the mutant rat strains at the locus -502 (Figure 5).

## Discussion

The fundamental feature of the epigenetic markers is their dynamic flux, which is the basic mechanism of environmental adaptability [12]. Methylation of CpG sites have been widely observed in vertebrates and occasionally reported in invertebrates [13]. Regulatory motifs on promoters can be identified by virtue of their preferential location relative to the TSS. The changes in methylation status of the CpG islands overlapping gene promoter enable epigenetic control of transcription. At the same time, established methylation pattern has to be maintained with high fidelity since aberrant methylation events (hypo- and hyper-methylation) lead to dysregulation of the downstream gene [14]. Methylation of DNA essentially leads to a repression of transcription by interfering with the binding sequence of transcription factors and through the binding of methyl-CpG binding proteins [15].

A comprehensive *insilico* analysis of epigenetic properties of the rat leptin gene promoter DNA sequence necessitates experimental testing that affords direct measurement of the methylation state of this promoter region. In the present study, 550bp length leptin gene promoter DNA sequence was analysed and identified four CpGs which were in close proximity to the TSS and had the potentials to be methylated. The present *in silico* analysis is in line with a study which noted that methylation surrounding the TSS is tightly linked to transcriptional silencing while methylation of more downstream regions is unassociated with the magnitude of gene expression [16,17]. Considering that the comparative genomics approach is widely used to identify functional sequence elements and regulatory networks [18] this analysis was performed as a prelude to the experimental testing of the leptin gene promoter methylation in WNIN mutant rat genomic samples which may help to design the experiments, to minimize possible experimental bias, and thus improve validity of the experimental data.

Leptin, a multifunctional hormone, is predominantly delivered by mature adipocytes. The present study demonstrated that the leptin mRNA expression levels were varying with their adipose levels in WNIN mutant rat strains i.e. WNIN/Ob & WNIN/GROb and their carrier and lean counterparts. The varying adipose tissue levels indicate that the genes associated with adipogenesis regulation may be different in these mutant WNIN rat phenotypes. These observations are in agreement with few reports which showed strong correlation between the levels of leptin mRNA expression and adipocyte size supporting the importance of leptin in altering the fat mass [19,20]. Another study on human subjects showed that the developmental increase in leptin mRNA expression in adipose tissue during childhood, reaching maximal capacity in adulthood [21]. In addition a study also depicted an evidence linking leptin to a direct regulation of adipose tissue metabolism through inhibition of lipogenesis and stimulation of lipolysis [22]. Thus these results support that the differentialexpression of leptin mRNA in these mutant phenotypes could be due to the genetic differences among the strains studied although these animals were fed with the same rat chow. According to our knowledge this is the first report on leptin mRNA expression in genetically mutant rat models originated from WNIN that follow the Mendelian genetic ratio’s (1:2:1). However the underlying mechanism for the close correlation between leptin mRNA level and adipocyte mass is not yet understood in these WNIN obese mutant rats.

Many researchers have suggested that the expression of genes controlling energy homeostasis could be regulated by epigenetic mechanisms, which may play a role in the development of obesity [23,24]. Considering this, the regulation of leptin gene expression in these rat models was studied by epigenetic approach i.e. by analysing promoter DNA methylation *in silico* and *in vivo* and correlated with gene expression. It was noticed that the leptin gene promoter DNA methylation percentages were varying in mutant WNIN obese rat phenotypes. The decreased percentage of methylation in leptin gene promoter in WNIN obese than in lean and carriers are in accordance with a recent study which reported genetic polymorphisms associated with differential methylation in the H19/IGF2 locus [25]. Another report indicates that genetic variation may have a substantial impact on local methylation patterns [26,27], but neither the extent to which methylation is affected by genetic variation, nor the mechanisms are yet clear. Our results also demonstrated that methylated CpGs are in close proximity to the TSS in the rat promoter studied. This work is in agreement with Melzner et al who showed that the three specific CpG sites within the minimal *LEP* promoter region played an important role in regulating reporter gene expression in human adipose cell line [11].

The present study clearly demonstrated that the body weights, adipocyte mass [28] and leptin gene expression are in line with leptin promoter DNA methylation percentages in WNIN obese mutant rats suggesting that the genetic differences can have profound influence on methylation patterns. The present *in silico* methylation prediction was in agreement with *in vivo* methylation status. The sole exception was at the locus -481 which showed methylation in *in vivo,* while the *in silico,* analysis of methylation could not predict. I*n silico* analysis also showed the presence of CpG at the locus -309 and its predicted methylation, which was identified proximal to the TSS in the rat promoter DNA sequence. Interestingly, our *in vivo* studies also demonstrated clearly that methylation was more at locus -309 which regulated leptin gene expression in WNIN mutant obese rat phenotypes. These observations are in line with several studies which showed that methylation of CpG sites that are close to the TSS in promoter DNA sequence will have more effect on regulation of gene expression [29]. Melzner et al. also reported that the CpGs in the proximal leptin promoter were highly methylated in pre-adipocytes and during maturation toward terminally differentiated adipocytes, this promoter region was found to be highly demethylated. Hence it was clear that the CpG sites which are close to TSS methylated may have profound effect on gene expression.

## Conclusion

The present study clearly demonstrated that the leptin gene expression is in line with leptin promoter DNA methylation patterns in WNIN obese mutant rats suggesting that genetic variation seemed to have an effect on methylation percentages and subsequently on the regulation of gene expression. These results may have implications for the functional interpretation of mechanisms underlying association of genetic variants with the development of obesity.

## Abbreviations

TSS: Transcription start site; WNIN: Wistar National Institute of Nutrition.

## Competing interests

The authors declare that they have no competing interests.

## Authors’ contributions

KRR, NVG and MR designed the study and drafted the manuscript. KRR, IJNP, AET, JS carried out experimental work. IJNP contributed for statistical analysis. All the authors read and approved the final manuscript.
